# Should I stay or should I go? Understanding families’ decisions regarding initiating, continuing, and terminating health services for managing pediatric obesity: the protocol for a multi-center, qualitative study

**DOI:** 10.1186/1472-6963-12-486

**Published:** 2012-12-31

**Authors:** Geoff DC Ball, Arnaldo Perez Garcia, Jean-Pierre Chanoine, Katherine M Morrison, Laurent Legault, Arya M Sharma, Rebecca Gokiert, Nicholas L Holt

**Affiliations:** 1 8B, Pediatric Centre for Weight and Health, Edmonton General Continuing Care Centre, 11111 Jasper Ave, Edmonton, AB, T5K0L4, Canada; 2 Room K4-212, Endocrinology and Diabetes Unit, British Columbia's Children's Hospital, 4480 Oak Street, Vancouver, BC, V6H3V4, Canada; 3 Department of Pediatrics & Population Health Research Institute, McMaster University, Hamilton, HSC 3A59, 1200 Main St. W, Ontario, L8N3Z5, Canada; 4 2300 rue Tupper, Montréal, Québec, H3H1P3, Canada; 5 406 CSC Royal Alexandra Hospital, Edmonton, AB, T5H3V9, CANADA; 6 Community-University Partnership, University of Alberta, Room 2-281 Enterprise Square, 10230 Jasper Avenue, Edmonton, AB, T5J4P6, Canada; 7 Faculty of Physical Education and Recreation, University of Alberta, P3-20S Van Vliet Centre, Edmonton, AB, T6G2H9, Canada

**Keywords:** Obesity, Pediatric, Treatment, Family, Qualitative, Attrition, Health services

## Abstract

**Background:**

At least two million Canadian children meet established criteria for weight management. Due to the adverse health consequences of obesity, most pediatric weight management research has examined the efficacy and effectiveness of interventions to improve lifestyle behaviors, reduce co-morbidities, and enable weight management. However, little information is available on families’ decisions to initiate, continue, and terminate weight management care. This is an important knowledge gap since a substantial number of families fail to initiate care after being referred for weight management while many families who initiate care discontinue it after a brief period of time. This research aims to understand the interplay between individual, family, environmental, and systemic factors that influence families’ decisions regarding the management of pediatric obesity.

**Methods/Design:**

Individual interviews will be conducted with children and youth with obesity (n = 100) and their parents (n = 100) for a total number of 200 interviews with 100 families. Families will be recruited from four Canadian multi-disciplinary pediatric weight management centers in Vancouver, Edmonton, Hamilton, and Montreal. Participants will be purposefully-sampled into the following groups: (*i*) *Non-Initiators* (5 families/site): referred for weight management within the past 6 months and did not follow-up the referral; (*ii*) *Initiators* (10 families/site): referred for weight management within the past 6 months and did follow-up the referral with at least one clinic appointment; and (*iii*) *Continuers* (10 families/site): participated in a formal weight management intervention within the past 12 months and did continue with follow-up care for at least 6 months. Interviews will be digitally recorded and analyzed using an ecological framework, which will enable a multi-level evaluation of proximal and distal factors that underlie families’ decisions regarding initiation, continuation, and termination of care. Demographic and anthropometric/clinical data will also be collected.

**Discussion:**

A better understanding of family involvement in pediatric weight management care will help to improve existing health services in this area. Study data will be used in future research to develop a validated survey that clinicians working in pediatric obesity management can use to understand and enhance their own health services delivery.

## Background

Few pediatric health issues have attracted as much attention in recent years as pediatric obesity. The volume of obesity-related research focused on epidemiology, etiology, and health consequences increased markedly over the past few decades. While many questions remain unanswered regarding how best to prevent and manage obesity in children, the magnitude of obesity and obesity-related health risks are clear. For instance, the Canadian Health Measures Survey [1] reported that the proportion of boys classified as overweight or obese increased from 14 to 31% between 1981 and 2009; among girls, excess weight increased from 14 to 25%. These trends represent dramatic increases for both boys (+120%) and girls (+79%). Longitudinal data from the US showed that the most striking changes in unhealthy weight gain in recent years have impacted children and youth who are already at high health risk because of their excess weight. Between 1999–2009, the proportions of individuals classified as overweight or obese remained relatively stable; however, trend analyses revealed a significant rise in the prevalence of *severe* obesity [2]. This change is clinically important since boys and girls with severe obesity are very likely to (*i*) remain obese into adulthood [3] and (*ii*) suffer from adverse metabolic health consequences including an increased risk of type 2 diabetes, cardiovascular disease, hypertension, nonalcoholic fatty liver disease, and the metabolic syndrome [4,5]. Beyond metabolic health, childhood obesity can negatively impact a number of other areas including physical function [1], psychosocial health [6-9], and life expectancy [10-12]. Viewed collectively, this body of evidence establishes pediatric obesity as a chronic condition that requires targeted, innovative approaches to reduce obesity-related health risks in order to optimize both quantity and quality of life [13].

Although the ideal weight management care model is not yet agreed upon, key principles have been described. Clinical practice guidelines [14] and expert recommendations [15-17] emphasize the value of taking a long-term, family-centred approach while combining lifestyle, behavioural, and cognitive techniques to improve dietary quality, increase physical activity, reduce physical inactivity, and improve psychosocial and familial health outcomes. These approaches can be used to describe most of the services offered by pediatric weight management centres across Canada [18]. Viewing obesity as a chronic care issue [19] is increasingly being used to guide weight management care [14,15]; this viewpoint encourages obesity-related health services to move *away from* a traditional, paternalistic framework, which was designed historically to manage acute health issues *towards* establishing a more collaborative, long-term partnership between clinicians and families, one that extends beyond the clinical setting to include community-based resources and supports. The Chronic Care Model (CCM) acknowledges the chronicity of obesity and commonalities between individuals regarding symptomatology, emotional impacts, lifestyle adjustments, and obtaining effective health care. Many families struggle with the physical, psychological, and social demands of obesity with limited help or support [20]. Most often, the help received (while well-intentioned) fails to optimize clinical care or meet families’ needs to effectively self-manage obesity. Consistent with the CCM, effective obesity management requires health service delivery that enables productive interactions between clinicians and families over time [21]. The well-established high levels of intervention attrition [22,23] and weight-related bias [24,25] indicate that researchers and clinicians must strive to better understand individuals’ and families’ experiences and improve obesity-related health services. A primary challenge for professionals providing health services for pediatric weight management relates to factors that influence families’ decisions regarding the initiation, continuation, and termination of care**.**

To our knowledge, limited data are available related to families’ reasons for not initiating weight management care; however, a number of surveys and medical record reviews have examined factors related to families’ attrition from pediatric obesity treatment. In one study [26], families who attended one or two visits at a multidisciplinary weight management clinic before discontinuing care reported unmet treatment needs, far distance from the clinic, scheduling conflicts, and a lack of medical insurance as primary reasons for their discontinuation. Another survey [27] showed that families that prematurely discontinued the first phase of an intensive behavioural treatment program did so because of excessive program length, lack of adequate transportation, unmet expectations, and their child’s desire to terminate. A large medical record review [28] examined attrition in children and youth with obesity who attended only one appointment at a specialized clinic and found that African-Americans and those with managed care insurance were more likely to dropout than Caucasians and those with indemnity insurance coverage. A second medical record review [29] compared children who did or did not finish a four-month intensive treatment program, which reported that non-completers were more likely to be Medicaid recipients, African-American, older, and self-report greater depressive symptomatology and lower self-concept than completers. One small Canadian study reinforces many of these findings. Of families that discontinued outpatient nutrition counselling for weight management after one appointment [30], parents’ reasons for non-return included clinic location, limited parking options, low satisfaction with the clinical environment, and counseling approach (e.g.,, intervention focus on the child rather than the family). While the aforementioned studies provide some insight into factors that impacted families’ termination of care, they also underscore the clear need for additional research to establish a stronger evidence base upon which to guide administrative and clinical decision-making related to pediatric weight management. This is especially important given that most of the research to date has been conducting in the US, which means issues that impact attrition such as health services funding and demographic characteristics are not generalizable to other countries.

With the aforementioned issues in mind, the overarching aim of the current study is to investigate the factors influencing families’ decisions regarding initiating, continuing, and terminating pediatric weight management care. This focus will allow our team to identify issues that can help to optimize the delivery of health services for managing pediatric obesity in Canada. Specifically, this research protocol was developed to answer to the following question: *After being referred for pediatric weight management, what micro (e.g., child, parent, family), meso (e.g., clinicians, clinic environment), and macro (e.g., health care system, environment) level factors are involved in families’ decisions regarding whether or not to initiate, continue, or terminate health services for managing pediatric obesity?*

The collective experience of our team suggests that approximately 50% of all boys and girls referred for specialized health services to manage pediatric obesity fail to attend an initial clinical appointment. By failing to initiate care, a substantial number of children miss out on opportunities for clinical evaluations and interventions that can (*i*) identify underlying medical, behavioural, and mental health issues and (*ii*) support families in making positive lifestyle changes. Developing a comprehensive understanding of families’ decisions regarding their initiation (or lack thereof) of health services will generate valuable data regarding whether potential barriers can be mitigated or opportunities can be enhanced to increase the likelihood that families initiate weight management care. Our real-world observations also suggest that among those families that do initiate care, most only do so for a short period of time. This issue is clinically relevant since long-term care enhances weight management success [14,15,31]. Families’ reasons foreither continuing or terminating care are likely to be complex, extend beyond simple, intrapersonal factors, and may not necessarily correspond to weight management success. The long-term continuation of care is important for weight loss maintenance, but also has important implications for health service delivery since a common challenge among pediatric weight management centres in Canada is limited resource availability (e.g., economic, personnel, time), which can limit treatment options [32].

## Methods/Design

### Methodology

Our research will use a multiple qualitative case study methodology [33]. Data will be collected from four distinct research sites with each of the following sites representing a *case*: (*i*) Pediatric Centre for Weight and Health in Edmonton, Alberta; (*ii*) Centre for Healthy Weights in Vancouver, British Columbia; (*iii*) Metabolism, Obesity and Health Program at McMaster Children’s Hospital, Hamilton, Ontario; and (*iv*) Healthy Weight Clinic in Montreal, Quebec. A case study approach is appropriate for this research since the topic of interest is represented within each selected case, and each case represents a population within the topic of interest [33]. Within each case, the *unit of analysis* will be the family members who were referred or received weight management care (e.g., a child/youth and a parent). By collecting perspectives from multiple families, we will be able to create an account that represents each of the four cases. Commonalities and differences between and within cases will then be identified. Ultimately, this will enable us to provide detailed information about experiences and decisions regarding families’ initiation, continuation, and termination of care that are relevant to centres across the country. The multiple qualitative case study methodology is appropriate for this study for several reasons. First, the research questions require detailed information about participants’ perceptions and experiences; such data could not be obtained through quantitative measures (e.g., questionnaires). Second, it is important to study multiple cases across Canada in order to establish local level variations in addition to common factors that influence families’ decisions to initiate, continue, and terminate care. Finally, case study methodology is appropriate for studying ‘bounded’ social systems. In the proposed study, each site is a bounded system with specific rules and norms of social interaction. Using this approach, researchers can obtain data to identify key issues relevant to each case (and shared between cases), triangulate key findings for interpretation, consider alterative explanations, and develop assertions about the cases [34,35]. The four multidisciplinary pediatric weight management centres represented in this study were selected based on their existing administrative and research infrastructure, national leadership roles of their program directors, diverse demographic characteristics of the communities they serve, and similar philosophical approaches to providing family-centred care.

### Study sample

A total of 100 families (n = 25/site) will be recruited for this study. The number of families per site has been estimated based on a previous study [36] in which an adequate level of saturation was reached after interviewing families at one weight management centre. The inclusion of a broader sample usually requires additional data to achieve saturation than would be required with a more tightly defined sample [37]. A purposeful sampling approach will be used, a strategy that is designed to identify the most information-rich cases from which to learn about issues that are fundamental to the purpose of the research [38]. Given the aims of the study, families will be recruited based on whether they satisfy the following eligibility criteria:

1. *Non-Initiators* (n = 5 families/site; 20 families total): Families in this group will include a child with obesity who was referred for weight management within the past six months, but did not follow-up the referral by attending a clinical appointment. We anticipate this group of families will be more difficult to recruit and enroll, which is reflected in the smaller sample size. This group of families will allow us to explore issues related to decisions regarding initiation of care.

2. *Initiators* (n = 10 families/site; 40 families total): Families in this group will include a child with obesity who was referred for weight management within the past six months, who did follow-up the referral by attending at least one clinical appointment, and discontinued care following a brief period of time. This group of families will allow us to explore issues related to both the initiation and premature termination of care.

3. *Continuers* (n = 10 families/site; 40 families total): Families in this group will have a child with obesity who was referred for weight management and completed a formal weight management intervention within the past 12 months and will have continued with follow-up care for at least six months. This group of families will allow us to explore issues related to both the initiation and continuation of care.

### Inclusion criteria

Families will be eligible for this study if children with obesity were (*i*) referred for weight management to one of the four multidisciplinary pediatric weight management centers, (*ii*) 10–17 years old at the time of referral, and (*iii*) possessed an age- and sex-specific body mass index (BMI) ≥97th percentile at the time of referral. Parents (mothers, fathers, and legal guardians) will be eligible for this study if they self-identify as the primary caregiver of a child with obesity. Families will determine the adult who can, in their view, best represent their family’s experiences and perceptions regarding pediatric weight management.

### Recruitment

To enhance our ability to successfully recruit families into all three categories from all four sites, we will apply three main strategies. First, we will offer families the option of holding interviews at times (e.g., evenings, weekends) and locations (e.g., weight management center, family residence) that are most convenient for them. Second, we will offer each family a $100 gift card at a local business or shopping mall as an incentive to participate and acknowledgement of the time, effort, and (potential) time away from work. Finally, we will work collaboratively across our four sites to enhance recruitment. At study initiation, we will develop a site-specific recruitment timeline, which will include milestones and a clear plan to document all successful and unsuccessful recruitment approaches. We will collectively discuss and develop study promotional materials and family recruitment letters, so similar information (both in English and French) will be used across study sites. Families in the *Non-Initiator* and *Initiator* groups will be contacted by regular mail with additional correspondence by telephone or e-mail, when appropriate, because they will not be attending regular clinic appointments. Following institutional ethics approvals, mailing addresses for potential participants will be retrieved from patient registries that are maintained by all four study centres. Families in the *Continuers* group will be contacted in-person by a member of each centre’s administrative staff during a scheduled clinical appointment. Study promotional materials will be shared with families at this time. If families express interest in the study, follow-up (either in-person, by telephone or email) for study recruitment will be initiated by research coordinators (RCs) at each site. Once inclusion criteria are confirmed, RCs will complete the informed consent and assent procedures with parents and children, respectively.

### Data collection

Participants (children and parents) will engage in one individual semi-structured interview each. Assuming that we interview one child and one parent per family, there will be a total of 200 interviews. Interviews will be conducted at each site by trained Graduate Research Assistants (GRAs) and/or RCs. Interviews will be 30 (child) to 60 (parent) minutes in length and will be digitally recorded using Olympus Digital Voice Recorders (WS-400S). Interviews will include open-ended questions about factors related to the initiation, continuation, and/or termination of care, reasons for making those decisions, who was involved in making those decisions, and how they feel about those decisions. Information about the micro (e.g., child, parent, family), meso (e.g., clinicians, clinical setting), and macro (e.g., health care system, environment) factors will be gathered; probes regarding perceptions, experiences, examples, and preferences related to decision-making will be used. Preferences, perceived strengths and limitations, and areas for improvement regarding health services for managing pediatric obesity will be explored. Participants will be asked to provide specific examples of challenges and opportunities they faced. A closing discussion will probe perceptions of need for long-term support, how support should be delivered, and expectations for maintaining changes initiated during and following treatment. Demographic and anthropometric/clinical information will also be obtained for contextual purposes. Demographic variables will include mailing address (to calculate geographic distance between weight management centers and families’ residences), dates of birth, sex, relationship between child and parent, ethnicity, and socioeconomic status. Anthropometric/clinical variables will include weight, height, waist circumference, BMI, BMI percentile (child only), BMI z-score (child only), presence/absence of obesity-related co-morbidities, and family history of chronic disease. Portable medical scales, stadiometers, and tape measures will be used to collect up-to-date weight, height, and waist circumference data, respectively, from all participants, which will be particularly important when interviews are conducted away from clinical settings. Demographic and clinical data will be collected by site-specific GRAs and RCs from several sources including weight management referral forms and research or medical charts for boys and girls who attended one of our centres. The accuracy and completeness of data gleaned from referral forms and charts will be confirmed with families upon study enrolment. Methodological rigour will be enhanced by adhering to evidence-based protocols for medical record review research [39,40].

### Data analysis

Digitally-recorded interview data from all centres will be submitted electronically to the *Comma Police* ( http://www.commapolice.com) for transcription. Subsequently, transcribed data will be entered verbatim into *N-VIVO 8* (QSR, Melbourne, Australia) for data management and analysis. Interviews held in Vancouver, Edmonton, and Hamilton will be conducted in English whereas interviews in Montreal will be conducted in either English or French. Data will be transcribed to text in the original language of the interview (English or French). At the final stage of analysis, results from both languages will be compared and contrasted. Data analysis will commence as soon as the first transcripts are received. Initial coding will be performed by GRAs and RCs at each of the four sites under the guidance of the principal and co-investigators. Although all data will be subjected to the same coding procedures (described below), analysis will be conducted on a case-by-case basis to identify specific issues based on participants’ experiences from each of the four research sites. Stake’s [34] categorical aggregation method for case study methodology will be used. First, transcripts will be read using line-by-line coding to identify specific themes. This process requires examining, questioning, and corroborating themes throughout the analysis. As themes are identified, similar instances or occurrences will be aggregated to create a basic coding schema. A written description will then be constructed to explain each category. During the final stages of coding, data from each center will be aggregated to establish common themes and specific local variations.

Consistent with case study methodology [34], several techniques have been built into the research design to enhance the methodological rigor of the analysis. First, obtaining data from multiple sources (children and parents) across four sites will allow us to triangulate the findings [34,38]. Second, data analysis will be corroborated by the research team who will compile a quarterly report of the findings to date, which will be discussed with all team members during quarterly team meetings. This process will also be repeated with the final results, which will allow us to corroborate the analysis and remain sensitive to local, contextual issues. Third, a member checking protocol [41] will be used. Participants in each of the four research sites will receive a summary of the findings and be asked to evaluate whether the analysis reflects their personal experiences in pediatric weight management care. Finally, the sample size is substantial and will enhance our ability to attain data saturation, which will allow us to draw meaningful conclusions from the data.

Information retrieved from referral forms and medical charts will include contextual data from children and parents. Data will be retrieved from these sources (and supplemented by information collected from families during individual interviews) using standardized case report forms that will be developed with input from all team members. Quantitative data will be analyzed using *SPSS 17.0* (SPSS Inc.; Chicago, IL). Continuous variables will be described by univariable summaries (e.g., means, medians, ranges, SDs) and frequency distributions will be determined for categorical variables. For key outcome variables, 95% confidence intervals will be reported for means and proportions. To display continuous variables, box plots and histograms will be used; bar charts will be used for categorical variables.

## Discussion

Childhood obesity has emerged as a priority health concern in Canada, but the manner with which health services are delivered for its management has received very little research attention. There is a clear need to gain a better understanding of how health services can be optimized for children with obesity (and their families) given that most research in this area has examined intervention efficacy and effectiveness, foci that leave several knowledge gaps remaining. A key research issue with relevance to pediatric weight management care is family engagement; in its absence, even the most effective intervention will be unlikely to bring about positive changes in health outcomes. In response to this lack of evidence, the present study is designed to understand the variety of factors at multiple levels that influence families’ decision regarding initiation, continuing, and terminating the management of pediatric obesity.

Some information exists related to factors that predict or influence families’ decisions to *terminate* pediatric weight management care (attrition) [42]. Study findings have been mixed with respect to factors that predict attrition (e.g., children’s BMI, sex), but practical issues including scheduling difficulties and services not meeting families expectations have been reported as common reasons for terminating care. It is noteworthy that most of the evidence on attrition is derived from US-based research, which limits generalizability to other countries given demographic and health care system differences. For instance, insurance coverage (or lack thereof) may influence attrition in the US, but in jurisdictions with universal health care coverage (e.g., Canada, United Kingdom, Australia, New Zealand), other factors are likely to be more salient. In addition, any differences in attrition that exist along racial or ethnic lines are likely to vary between nations given inter-country differences in cultural diversity and immigration patterns. These observations reinforce the importance of acquiring attrition-related data from health care settings beyond the US.

In contrast to the growing body of literature on attrition, currently, there is scant information available on issues related to families’ decisions regarding their *initiation* of pediatric weight management care. Determinants of non-initiation are of particular importance given that, in our team’s clinical experience, a substantial number of children and families fail to initiate care, which includes choosing to not follow-up their clinician’s referral to a weight management clinic or deciding to not self-refer themselves into clinic- or community-based services. Such decisions mean that families miss out on opportunities to benefit from weight management care, which can include (*i*) identifying underlying obesity-related co-morbidities (e.g., hypertension, dyslipidemia, fatty liver disease) that may require additional specialized health services and (*ii*) participating in family- and lifestyle-based interventions that are known to be effective in helping children with obesity (and their families) to improve their weight and health [43].

By applying a qualitative research approach, we will obtain rich, contextual information across our four study sites that cannot be gleaned from brief surveys or medical record reviews. Qualitative methodology provides an excellent means of understanding the meaning behind reports that have linked attrition to parents’ perceptions of quality of care and unfulfilled treatment needs [42]. Our decision to include both children and parents is noteworthy since we cannot assume that factors which determine the initiation, continuation, and termination of care are similar for all family members. Given differences in age and stage, salient concerns for parents (e.g., current or future medical health risks) may not resonate with children (and *vice versa*). Further, this study also acknowledges the roles that children play in their self-management, a key feature of managing obesity as a chronic condition. Building on research to date, our study will investigate families’ perceptions, experiences, and needs at both ends of the treatment continuum (when it is initiated and when it is discontinued). Speaking with families at different levels of motivation to change lifestyle and behavioural issues and with unique experiences in clinical weight management will enable our team to better understand how to optimize health services for managing pediatric obesity.

Through our search of the literature and collaborative efforts in developing the current research, we identified gaps in evidence that are demanding new theoretical and methodological developments. Consequently, psychosocial factors that affect initiation, continuation, and termination of pediatric weight management care will not only be defined, but also understood to which a grounded theory of parental involvement in different stages of pediatric management care will be developed. The study is also ideally-suited for instrument building and data triangulation [44]. Interview and contextual (e.g., anthropometry, demo-graphy) data will be used to develop two new surveys (one for parents, one for children) that will be developed, piloted, and validated in follow-up research. These tools will be broadly available for researchers, clinicians, and decision-makers to administer in order to study (as well as mitigate or manage) factors related to the initiation, continuation, and termination of pediatric weight management care. Ultimately, this information can inform how health services should evolve to better meet the needs of children with obesity and their families.

## Competing interests

In relation to the present research, all authors are in agreement that they have no financial or non-financial interests to declare.

## Authors’ contributions

GDCB conceived of the study and co-authored the first draft of the manuscript (with APG), APG co-authored the first draft of the manuscript (with GDCB), JPC assisted with study design as well as writing and editing the manuscript, KMM assisted with study design as well as writing and editing the manuscript, LL assisted with study design as well as writing and editing the manuscript, AMS assisted with study design as well as writing and editing the manuscript, RG assisted with study design as well as writing and editing the manuscript, NLH assisted with study design as well as writing and editing the manuscript. All authors have read and approved the final manuscript.

## Pre-publication history

The pre-publication history for this paper can be accessed here:

http://www.biomedcentral.com/1472-6963/12/486/prepub
